# Effects of New Mutations in *BMPRIB*, *GDF9*, *BMP15*, *LEPR*, and *B4GALNT2* Genes on Litter Size in Sheep

**DOI:** 10.3390/vetsci10040258

**Published:** 2023-03-28

**Authors:** Xuewen Ji, Ziwei Cao, Qi Hao, Mei He, Ming Cang, Haiquan Yu, Qing Ma, Xihe Li, Siqin Bao, Jianguo Wang, Bin Tong

**Affiliations:** 1 The State Key Laboratory of Reproductive Regulation and Breeding of Grassland Livestock, School of Life Sciences, Inner Mongolia University, Hohhot 010070, China; 2 Xilingol Mengzhiyuan Animal Husbandry Company, Xilingol 026000, China; 3 Institute of Animal Sciences, Ningxia Academy of Agricultural and Forestry Sciences, Yinchuan 750001, China

**Keywords:** *BMP15*, *BMPR1B*, *B4GALNT2*, *GDF9*, *LEPR*, litter size, sheep

## Abstract

**Simple Summary:**

*BMPRIB*, *GDF9*, *BMP15*, *LEPR*, and *B4GALNT2* genes were regarded as leading candidates for reproductive traits in sheep. Our recent studies identified 13 novel mutations associated with litter size in the Mongolia sheep breed in these 5 genes. In this study, we performed an association analysis of the 13 new mutations and 7 known ovine prolificacy-related mutations with litter size in Ujimqin, the F1 population of Dorper × Ujimqin crossbred, and the F1 population of Suffolk × Ujimqin crossbred. The results suggested that *BMPR1B* c.746A>G (*FecB*), *GDF9* c.994A>G (*FecG^A^*), and *BMP15* c.31_33CTTinsdel (*B1*) may be potentially effective genetic markers to improve the litter size in sheep.

**Abstract:**

Prolificacy is a crucial characteristic of livestock, particularly for species such as sheep that have many births. The objectives of this study were as follows: (1) to investigate the genetic diversity of the 13 new and 7 known variants in the *BMPRIB*, *GDF9*, *BMP15*, *LEPR*, and *B4GALNT2* genes in Ujimqin (UM), the F1 population of Dorper × Ujimqin crossbred (DPU), the F1 population of Suffolk × Ujimqin crossbred (SFKU), Sonid sheep (SN), Tan sheep (Tan), Hu sheep (Hu), and Small-tailed Han sheep (STH) sheep breeds/populations; (2) to perform an association analysis of the above 20 variants with litter size in 325 UM, 304 DPU, and 66 SFKU sheep populations; (3) to compare the frequencies of the litter-size-related alleles of these 20 variants among 8 sheep breeds/populations (the above seven sheep breeds + Mongolia sheep breed). With the use of the Sequenom MassARRAY^®^SNP assay technology, these 20 mutations were genotyped. The association analysis results showed that the c.746A>G (*FecB*) mutation in *BMPR1B* was significantly associated with the litter size of UM and DPU, the c.994A>G (*FecG^A^*) in *GDF9* was significantly associated with the litter size of SFKU, and the c.31_33CTTinsdel (*B1*) in *BMP15* was significantly associated with the litter size of UM. Our findings might provide valuable genetic markers for expanding sheep litter sizes.

## 1. Introduction

Ujimqin sheep (UM, Ovis aries), an old and primitive sheep breed, are distributed mainly in grassland areas in northern China and southern Mongolia and belong to Mongolia sheep (MG), which serve as the ancestor of several short, fat-tailed sheep breeds in China, including UM, Sonid (SN), Hulunbuir, Tan, Duolang sheep, Bayanbulak, Small-tailed Han (STH), and Hu sheep [1]. They have fat-tailed, generate top-notch meat and carpet wool, have a good body conformation, a powerful gait, and an impressive adaption to a variety of ecological settings [1,2,3]. The lamb of UM has recently become increasingly popular in China and has been acknowledged as a greenery food. The production rate of UM is constrained, nonetheless, because of seasonal estrus and a low prolificacy (the mean litter size was 1.03~1.13).

Reproductive features such as ovulation frequency and litter size have a significant impact on the sheep industry. Traditional breeding techniques have not been successful in rapidly increasing litter size in sheep because of the low heritability of litter size. However, to efficiently enhance litter size in sheep, a better technology is marker-assisted selection (MAS), which chooses animals with improvements in crucial features during a brief period of time at a minimal cost [4]. There has been a lot of research conducted on genetic diversity in sheep litter size to date. Breeds range significantly from one another, and even within breeds and sub-breeds, there are many variances. Except for the well-known variants including the *BMPRIB* (*FecB^B^*), *BMP1*5 (*FecX^B^*, *FecX^G^*, *FecX^H^*, *FecX^I^*, *FecX^L^*, *FecX^O^*, *FecX^R^*, *FecX^Bar^*, and *FecX^Gr^*), *GDF9* (*FecG^A^*, *FecG^E^*, *FecG^F^*, *FecG^H^*, *FecG^1^*, *FecG^T^*, and *FecG^V^*), *B4GALNT2* (*FecL^L^*), and *LEPR* (*FecD^D^*) genes (summarized in Table 1 in [5]), our recent studies found many novel mutations associated with litter size in the MG sheep population, such as the g.46544883A>G in the 3′ untranslated region (3′ UTR), the c.1040T>C (Phe347Ser) in the exon 2, the g.46547859C>T in the promotor of *GDF9* [5], and the g.46547645T>G in the promotor of *GDF9* [6]; the c.1470G>T (490Thr) in the exon 10 of *BMPR1B* [7]; the g.509807863G>A in the promotor of *BMP15* [8]; the c.240C>T (80Asn) and c.279C>T (93Ser) in the exon 2 of *LEPR* [9]; and the g.25929637G>A, g.25929679T>C, g.25929819A>G, and g.25929965A>T variants in the intron 7 of *B4GALNT2* [10]. Thus, an investigation of the markers which were associated with litter size in the MG breed will provide potentially useful genetic markers for breeding in the UM breed due to the related close genetic background.

Hence, the following were the goals of the current study: (1) to investigate the genetic diversity of the 13 new variants and 7 known variants in the *BMPRIB*, *GDF9*, *BMP15*, *LEPR*, and *B4GALNT2* genes in a total of 969 individuals of 7 sheep breeds/populations, UM, the F1 population of Dorper × Ujimqin crossbred (DPU), and the F1 population of Suffolk × Ujimqin crossbred (SFKU), SN, Tan, Hu, and STH sheep breeds/populations; (2) to perform an association analysis of the 13 new variants and 7 known variants in the above 5 genes with a litter size of 325 UM, 304 DPU, and 66 SFKU populations; and (3) to compare the frequencies of the litter-size-related alleles of these variants among eight sheep breeds/populations, UM, DPU, SFKU, MG [5,6,7,8,9,10], SN, Tan, Hu, and STH.

## 2. Materials and Methods

### 2.1. Animals

A total of 695 sheep, including 325 purebred UM ewes, 304 DPU ewes, and 66 SFKU ewe populations, were sampled from Xilingol Mengzhiyuan Animal Husbandry Co., Ltd. of the Inner Mongolia of China. The sheep were all kept in similar environments with unrestricted food and water access. In 2021, the UM, DPU, and SKFU ewes’ litter sizes were recorded. The mating of all of these UM, DPU, and SKFU ewes occurred naturally in the fall of 2020 when they were two years old. For the UM, DPU, and SFKU ewes, the ewes were randomly mated by more than 15 Ujimqin sires, more than 15 Dorper sires, and three Suffolk sires, respectively. There was no mating record. No particular ram was employed for mating.

In addition, 30 Tan ewes (a low prolific breed) were collected from Yanchi of Ningxia province, 30 STH (a highly prolific breed) ewes, and 30 Hu (a highly prolific breed) ewes were collected from Zhengzhou of Henan Province, and 184 SN (a low prolific breed) ewes were collected from the Left Sonid Banner of Inner Mongolia, China. Within each sheep breed, there was no clear preference for a particular father or maternal grandfather of the ewes, and the animal panel for each breed probably represents a random sample of the population of each sheep breed. The details of the seven sheep breeds are displayed in Table 1.

Each sheep provided ten milliliters of blood, which was used for genotyping and variation sequencing. Using a TIANamp Blood DNA kit (TIANGEN Biotech, Beijing, China), 416 sheep’s genomic DNA samples were extracted from blood samples. Using an agarose gel electrophoresis machine and a Nanodrop^®^ spectrophotometer (Thermo Fisher Scientific, Waltham, MA, USA), the quantity and quality of the isolated DNA were assessed.

### 2.2. SNP Genotyping by iPLEX MassARRAY

The newly identified 12 mutations in our recent studies, including the c.1470G>T in *BMPR1B* [7]; the g.46547859C>T, c.1040T>C, and g.46544883A>G in *GDF9* [5]; the g.46547645T>G in *GDF9* [6]; the g.509807863G>A in *BMP15* [8]; the c.240C>T and c.279C>T in *LEPR* [9]; and the g.25929637G>A, g.25929679T>C, g.25929819A>G, and g.25929965A>T in *B4GALNT2* [10], as well as seven known mutations including the c.746A>G (*FecB*) in *BMPR1B* [11,12,13]; the c.260G>A (*FecG^1^*) [14] and c.994A>G (*FecG^A^*) in *GDF9* [14]; the c.31_33CTTinsdel (*B1*) and c.755T>C (Lue252Pro) in *BMP15* [14,15]; the c.185G>A (*FecD*) in *LEPR* [16]; and the c.440C>T and c.823C>T in *B4GALNT2* [17], were genotyped with the MassARRAY^®^ SNP genotyping system (Agena Bioscience, San Diego, CA, USA) in the 325 UM, 30 DPU, 66 SFKU, 184 SN, 30 Tan, 30 Hu, and 30 STH sheep populations. Assay Design Suite (http://agenabio.com/assay-design-suite-20-software, 24 June 2022) was used to design PCR and extension primers from the sequences, including each target mutation and around 100 upstream and downstream bases. With the help of the Sequenom MassARRAY iPLEX technology, the genotype of each SNP was examined [18]. The software MassARRAY Typer 4.0 Analyzer (Agena Bioscience) was used to examine the obtained data.

### 2.3. Statistical Analyses

Hardy–Weinberg equilibria, genotypic, and allelic frequencies were computed for the UM, DPU, SFKU, SN, Tan, Hu, and STH sheep populations. Nei’s methods were used to calculate the population genetic indicators, expected heterozygosity (He), observed heterozygosity (Ho), effective allele numbers (n_e_), and the polymorphism information content (PIC). [19]. Using a χ^2^ test, the allelic frequency within each mutation was analyzed. A two-way chi-squared test was used to examine the genetic influences of each SNP on the litter size of the UM, DPU, and SFKU alleles [7,8,9]. Their associations and effects could not be accurately evaluated when the proportion of sheep with a certain genotype was less than ten. Therefore, animals carrying this genotype were not included in the study, except for *FecB* mutation.

## 3. Results

### 3.1. Genetic Diversity Analysis

Tables S1–S5 list the frequencies of the two alleles, three genotypes, and genetic indices for each variant in the UM, DPU, SFKU, Tan, Hu, and STH sheep populations (Ho, He, n_e_, and PIC).

Significant departures at the 5% level were detected at the c.746A>G site of *BMPR1B* in STH (Table S1), at the g.46547645T>G and c.1040T>C (Phe347Ser) sites of *GDF9* in MG (Table S2), at the c.755T>C (Lue252Pro) site of *BMP15* in SN and MG (Table S3), at the c.185G>A (Arg62His) site of *LEPR* in MG (Table S4), at the c.440C>T site of *B4GALNT2* in Tan, at the g.25929679T>C site of *B4GALNT2* in SN and MG, and at the g.25929965A>T site of *B4GALNT2* in SFKU and SN populations (Table S5).

There was no polymorphism at the c.746A>G site of *BMPR1B* in SFKU (Table S1), at the c.994A>G site of *GDF9* in Tan and Hu, at the c.1040T>C site of *GDF9* in UM, SFKU, Tan, Hu, and STH (Table S2), and at the g.509807863G>A site of *BMP15* in Tan, Hu, and STH populations (Table S3). The values of the PIC of the c.746A>G (Gln249Arg) of *BMPR1B* presented with related low polymorphism in UM, DBU, MG, and Tan sheep, and moderate polymorphism in Hu and STH sheep populations (Table S1). The values of the PIC of the c.1470G>T (490Thr) of *BMPR1B* presented with related moderate polymorphism in UM, DBU, SFKU, SN, MG, and Tan sheep, and low polymorphism in the Hu and STH sheep populations (Table S1). The values of the PIC of the g. 46547859C>T of *GDF9* presented with related low polymorphism in UM, SFKU, SN, MG, Tan, Hu, and STH sheep, and moderate polymorphism in DBU sheep (Table S2). The values of the PIC of the g.46547645T>G of *GDF9* presented with related moderate polymorphism in UM, DBU, SN, Hu, and STH sheep, and low polymorphism in the SFKU, MG, and Tan sheep populations (Table S2). The values of the PIC of the c.260G>A (Arg87His), c.994A>G (Val322Ile), and c.1040T>C (Phe347Ser) of *GDF9* presented with related low polymorphism in other sheep populations (Table S2). The values of the PIC of the g. 46544883A>G of *GDF9* presented with related moderate polymorphism in DBU sheep, and low polymorphism in other sheep populations (Table S2). The values of the PIC of the g.509807863G>A of *BMP15* presented with related low polymorphism in eight sheep populations (Table S3). The values of the PIC of the c.31_33CTTinsdel (11Leu deletion) of *BMP15* presented with related moderate polymorphism in eight sheep populations (Table S3). The values of the PIC of the c.755T>C (Lue252Pro) of *BMP15* presented with related moderate polymorphism in STH sheep, and low polymorphism in other sheep populations (Table S3). The values of the PIC of the c.185G>A (Arg62His) of *LEPR* presented with related low polymorphism in UM, DBU, MG, Tan, and STH sheep, and moderate polymorphism in SFKU, SN, and Hu sheep populations (Table S4). The values of the PIC of the c.240C>T (80Asn) and c.279C>T (93Ser) of *LEPR* presented with related moderate polymorphism in eight sheep populations (Table S4). The values of the PIC of the c.440 C>T (Pro160Leu) of *B4GALNT2* presented with related moderate polymorphism in Hu sheep, and low polymorphism in other sheep populations (Table S5). The values of the PIC of the g.25929637G>A and g.25929965A>T of *B4GALNT2* presented with related low polymorphism in eight sheep populations (Table S5). The values of the PIC of the g.25929679T>C of *B4GALNT2* presented with related low polymorphism in UM, DBU, Tan, Hu, and STH sheep, and moderate polymorphism in SFKU, SN, and MG sheep populations (Table S5). The values of the PIC of the g.25929819A>G of *B4GALNT2* presented with related low polymorphism in UM, DBU, Hu, and STH sheep, and moderate polymorphism in SFKU, SN, MG, and Tan sheep populations (Table S5). The values of the PIC of the c.823C>T (Pro288Ser) of *B4GALNT2* presented with related low polymorphism in DBU, SFKU, SN, Tan, and STH sheep, and moderate polymorphism in UM, MG, and Hu sheep populations (Table S5).

### 3.2. Associations between Mutations in Candidate Genes and Litter Size in Sheep

#### 3.2.1. Associations between Mutations in BMPR1B and Litter Size in Sheep

In 325 UM, 304 DPU, and 66 SFKU ewes, the effects of the newly discovered c.1470G>T (490Thr) mutation related to the size of MG litters and the well-known c.746A>G (*FecB*) variant on litter size were examined (Table 2). For c.746A>G (*FecB*), the litter size of UM and DPU ewes with the AG genotype were significantly higher (*p* < 0.05) than those of ewes with the AA genotype, although the sample size of the AG genotype in UM and DPU was small (Table 2). For c.1470G>T, there was no significant difference in litter size between any two genotypes in each sheep population (Table 2).

**Figure 1:**
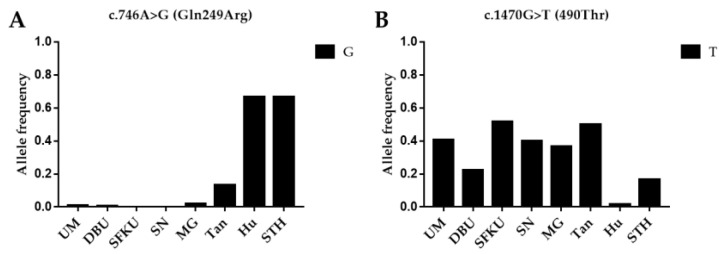
Distribution of two *BMPR1B* mutations allele frequencies in various sheep breeds/populations. (**A**) The G allelic frequencies of c.746A>G (*FecB*) in *BMPR1B* in eight sheep breeds/populations. (**B**) The T allelic frequencies of c.1470G>T in *BMPR1B* in eight sheep breeds/populations. UM: Ujimqin sheep, DPU: Dorper × Ujimqin F1 population, SFKU: Suffolk × Ujimqin F1 population, SN: Sonid sheep, MG: Mongolia sheep, Tan: Tan sheep, Hu: Hu sheep, STH: Small-tailed Han sheep. The results of χ^2^ tests for two mutations between any two sheep breeds/populations are listed in Table S6.

To determine the frequency of alleles linked with litter size in various sheep breeds/populations, the G allele of c.746A>G (*FecB*) and the T allele of c.1470G>T in *BMPR1B* were compared in the UM, DPU, SFKU, SN, MG, Tan, Hu, and STH sheep populations. Hu and STH had significantly higher allele frequencies of the G allele in the c.746A>G (*FecB*) mutation than other breeds (Figure 1A and Table S6). In contrast, Hu and STH sheep breeds had much lower T allele frequencies of c.1470G>T than practically all other sheep breeds (Figure 1B and Table S6).

#### 3.2.2. Associations between Mutations in GDF9 and Litter Size in Sheep

The effects of the new g.46547859C>T, g.46547645T>G, c.1040T>C (Phe347Ser), and g.46544883A>G mutations in *GDF9*, and the known c.260G>A (*FecG^1^*) and c.994A>G (*FecG^A^*) variants of *GDF9* on litter size were analyzed in 325 UM, 304 DPU, and 66 SFKU ewes (Table 3). For c.994A>G (*FecG^A^*), the litter size of SFKU ewes with the AG genotype was significantly higher (*p* < 0.05) than that of ewes with the AA genotype (Table 3).

**Figure 2:**
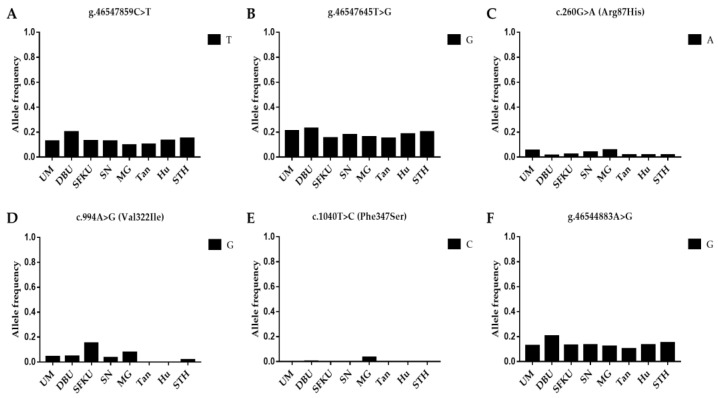
Distribution of six *GDF9* mutations’ allele frequencies in various sheep breeds/populations. (**A**) The T allelic frequencies of g.46547859C>T in *GDF9* in eight sheep breeds/populations. (**B**) The G allelic frequencies of g.46547645T>G in *GDF9* in eight sheep breeds/populations. (**C**) The A allelic frequencies of c.260G>A (*FecG^1^*) in *GDF9* in eight sheep breeds/populations. (**D**) The G allelic frequencies of c.994A>G (*FecG^A^*) in *GDF9* in eight sheep breeds/populations. (**E**) The C allelic frequencies of c.1040T>C in *GDF9* in eight sheep breeds/populations. (**F**) The G allelic frequencies of g.46544883A>G in *GDF9* in eight sheep breeds/populations. UM: Ujimqin sheep, DPU: Dorper × Ujimqin F1 population, SFKU: Suffolk × Ujimqin F1 population, SN: Sonid sheep, MG: Mongolia sheep, Tan: Tan sheep, Hu: Hu sheep, STH: Small-tailed Han sheep. The results of χ^2^ tests for two mutations between any two sheep breeds/populations are listed in Table S7.

The litter-size-associated T allele of g.46547859C>T, G allele of g.46547645T>G, A allele of c.260G>A (*FecG^1^*), G allele of c.994A>G (*FecG^A^*), C allele of c.1040T>C (Phe347Ser), and G allele of g.46544883A>G in *GDF9* were compared in the UM, DPU, SFKU, SN, MG, Tan, Hu, and STH sheep populations. The distribution of the litter-size-associated alleles of each mutation exhibited a related low frequency in each sheep breed/population (Figure 2A–F), although there were some statistical differences among some sheep breeds/populations (Table S7). Among these alleles, the C allele of c.1040T>C, which is associated with the litter size of MG, was almost absent null in other sheep breeds/populations (Figure 2E).

#### 3.2.3. Associations between Mutations in BMP15 and Litter Size in Sheep

The effects of the new g.509807863G>A mutation and the known c.31_33CTTinsdel (*B1*) and c.755T>C (Lue252Pro) variants of *BMP15* on litter size were analyzed in 325 UM, 304 DPU, and 66 SFKU ewes (Table 4). For c.31_33CTTinsdel (*B1*), the litter size of UM ewes with the CCT.DEL genotype was significantly higher (*p* < 0.01) than that of ewes with the CTT.CTT genotype (Table 4).

**Figure 3:**
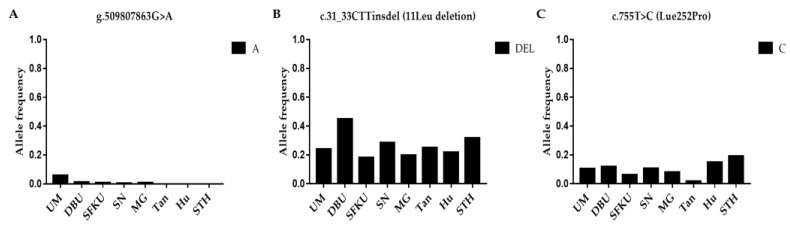
Distribution of three *BMP15* mutations’ allele frequencies in various sheep breeds/populations. (**A**) The A allelic frequencies of g.509807863G>A in *BMP15* in eight sheep breeds/populations. (**B**) The DEL allelic frequencies of c.31-33CTTinsdel (*B1*) in *BMP15* in eight sheep breeds/populations. (**C**) The A allelic frequencies of c.755T>C in *BMP15* in eight sheep breeds/populations. UM: Ujimqin sheep, DPU: Dorper × Ujimqin F1 population, SFKU: Suffolk × Ujimqin F1 population, SN: Sonid sheep, MG: Mongolia sheep, Tan: Tan sheep, Hu: Hu sheep, STH: Small-tailed Han sheep. The results of χ^2^ tests for two mutations between any two sheep breeds/populations are listed in Table S8.

The litter-size-associated A allele of g.509807863G>A, the A allele of c.755T>C in *BMP15*, and the DEL allele of c.31-33CTTinsdel (*B1*) was compared in the UM, DPU, SFKU, SN, MG, Tan, Hu, and STH sheep populations (Figure 3A–C and Table S8). The frequencies of the DEL allele in c.31-33CTTinsdel (*B1*) were significantly higher in DPU than in other breeds (Figure 3B and Table S8). The distributions of the A alleles of g.509807863G>A and the C allele of c.755T>C exhibited a related low frequency in each sheep breed/population (Figure 3A,C), although there were some statistical differences among some sheep breeds/populations (Table S8).

#### 3.2.4. Associations between Mutations in LEPR and Litter Size in Sheep

The effects of the new c.240C>T (80Asn) and c.279C>T (93Ser) mutations of *LEPR* and the known c.185G>A (*FecD*) of *LEPR* on litter size were analyzed in 325 UM, 304 DPU, and 66 SFKU ewes (Table 5). No statistically significant difference existed between mutation and litter size in our experimental UM, DPU, and SFKU sheep populations (Table 5).

**Figure 4:**
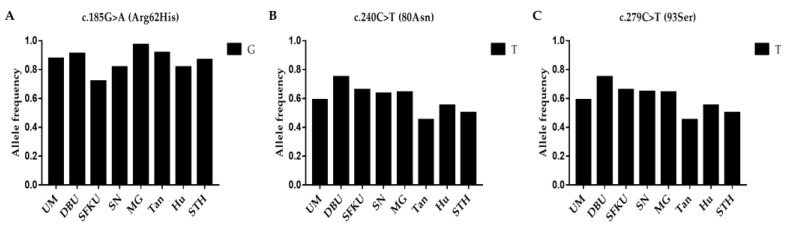
Distribution of three *LEPR* mutations’ allele frequencies in various sheep breeds/populations. (**A**) The G allelic frequencies of c.185G>A (*FecD*) in *LEPR* in eight sheep breeds/populations. (**B**) The T allelic frequencies of c.240C>T in *LEPR* in eight sheep breeds/populations. (**C**) The T allelic frequencies of c.279C>T in *LEPR* in eight sheep breeds/populations. UM: Ujimqin sheep, DPU: Dorper × Ujimqin F1 population, SFKU: Suffolk × Ujimqin F1 population, SN: Sonid sheep, MG: Mongolia sheep, Tan: Tan sheep, Hu: Hu sheep, STH: Small-tailed Han sheep. The results of χ^2^ tests for two mutations between any two sheep breeds/populations are listed in Table S9.

The litter-size-associated G allele of c.185G>A (*FecD*), the T allele of c.240C>T, and the T allele of the c.279C>T mutations in *LEPR* were compared in the UM, DPU, SFKU, SN, MG, Tan, Hu, and STH sheep populations (Figure 4A–C and Table S9). The litter-size-associated G allele of c.185G>A (*FecD*) exhibited a remarkably high frequency in each sheep breed/population (Table 4A). In addition, the T alleles of c.240C>T and c.279C>T, which related to the litter size of MG, were higher in DPU and lower in Tan sheep (Figure 4B,C and Table S9).

#### 3.2.5. Associations between Mutations in B4GALNT2 and Litter Size in Sheep

The effects of the new g.25929637G>A, g.25929679T>C, g.25929819A>G, and g.25929965A>T of *B4GALNT2*, and the known c.440C>T and c.823C>T mutations of *B4GALNT2* on litter size were analyzed in 325 UM, 304 DPU, and 66 SFKU ewes (Table 6). There was no significant difference between any mutation and litter size in our experimental UM, DPU, and SFKU sheep populations (Table 6).

**Figure 5:**
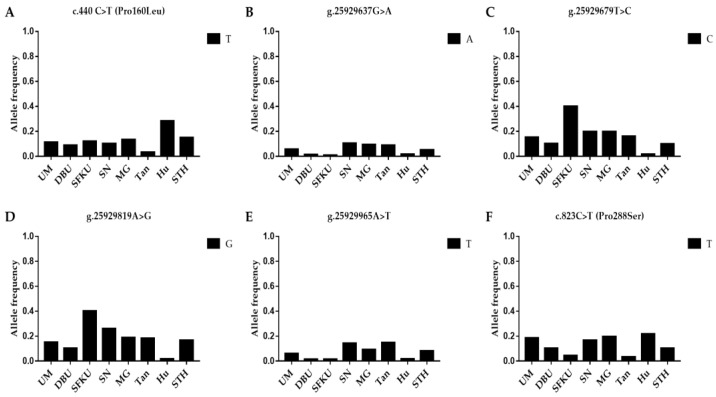
Distribution of six *B4GALNT2* mutations’ allele frequencies in various sheep breeds/populations. (**A**) The T allelic frequencies of c.440C>T in *B4GALNT2* in eight sheep breeds/populations. (**B**) The A allelic frequencies of g.25929637G>A in *B4GALNT2* in eight sheep breeds/populations. (**C**) The C allelic frequencies of g.25929679T>C in *B4GALNT2* in eight sheep breeds/populations. (**D**) The G allelic frequencies of g.25929819A>G in *B4GALNT2* in eight sheep breeds/populations. (**E**) The T allelic frequencies of g.25929965A>T in *B4GALNT2* in eight sheep breeds/populations. (**F**) The T allelic frequencies of g.46544883A>G in *B4GALNT2* in eight sheep breeds/populations. UM: Ujimqin sheep, DPU: Dorper × Ujimqin F1 population, SFKU: Suffolk × Ujimqin F1 population, SN: Sonid sheep, MG: Mongolia sheep, Tan: Tan sheep, Hu: Hu sheep, STH: Small-tailed Han sheep. The results of χ^2^ tests for two mutations between any two sheep breeds/populations are listed in Table S10.

The litter-size-associated T allele of c.440C>T, the A allele of g.25929637G>A, the C allele of g.25929679T>C, the G allele of g.25929819A>G, the T allele of g.25929965A>T, and the T allele of c.823C>T in *B4GALNT2* were compared in the UM, DPU, SFKU, SN, MG, Tan, Hu, and STH sheep populations (Figure 5A–F and Table S10). The frequency of the litter-size-associated T allele of c.440C>T was significantly higher in Hu sheep than in other sheep breeds/populations, except for STH sheep (Figure 5A and Table S10). The distribution of alleles of each mutation in *B4GALNT2* exhibited a related low frequency in each sheep breed/population (Figure 5A–F), although there were some statistical differences among some sheep breeds/populations (Table S10).

## 4. Discussion

In this study, we performed association analyses of the 13 newly identified mutations associated with the litter size of MG, and 7 known mutations in the *BMPRIB*, *GDF9*, *BMP15*, *LEPR*, and *B4GALNT2* genes with litter size in UM, DPU, and SFKU sheep breeds/populations. To the best of our knowledge, this is the first investigation of these 20 polymorphisms on litter size in UM, DPU, and SFKU sheep populations, and the litter-size-associated alleles of 20 polymorphisms were discussed for the first time for the distribution of allelic frequency among eight sheep breeds/populations, in addition to significant alleles for breeding and conservation in local lamb sheep breeds.

Several studies have shown that the *FecB* (c.746A>G) mutation in *BMPR1B* is associated with the reproductive traits in many sheep breeds (Booroola Merino [1,2,3,4,5,6,7,8,9,10,11,12,13], Javanese [20], small-tailed Han [21], Hu [22], Garole and Kendrapada [23], Kalehkoohi [24], and Wadi sheep [25]). In addition, numerous studies have focused on the polymorphisms of *BMPR1B* instead of *FecB*. A shift from C to A at position eight of the amplified *BMPR1B* exon segment influences the litter size of Mehraban sheep [26]. The T864C mutation in exon 9 of *BMPR1B* does not alter amino acids, but the genotype distribution of different litter size variables in ewes (singletons ewes, twin ewes, and multiples ewes) differs significantly [27]. In addition, the g.29362047T>C in the 5′ untranslated region and the g.29427689G>A SNP in exon 8 of *BMPR1B* had strong impacts on the litter size of Hu sheep [28]. Recent research indicates that a 10 bp insertion/deletion was significantly linked to the litter size of Australian White sheep [29]. Our recent study found a silent c.1470G>T (490Thr) mutation, in exon 10 of *BMPR1B* in MG, which affected litter size in MG, but it did not affect litter size in UM with a small sample size (a preliminary study, [7]). As was found in our preliminary study, there was no association in UM with a related big sample size in this study, and neither was there for DPU or SKFU. Although the silent c.1470G>T (490Thr) mutation was predicted to change the mRNA secondary structure of *BMPR1B* and increase the stability of the mRNA secondary structure by reducing minimum free energy, it is hypothesized that this mutation has a significant breed-specific impact on various sheep breeds. In addition, the G allele of *FecB* (c.746A>G) was the causative mutation for high prolificacy in Hu and STH sheep [30], showing a high allele frequency in this study. The majority of contemporary Chinese sheep breeds, including the low-prolific UM, SN, and Tan sheep, as well as the high-prolific STH and Hu sheep, have a connection to MG. [1]. The UM and SN sheep of Inner Mongolia’s grasslands, on the other hand, are genetically more similar to MG than they are to other species since they live in similar environments and have similar ancestries. In the UM, SN, and Tan sheep, the distribution of the *FecB* well-characterized G allele frequency was very low or null, which is similar to the findings of the MG ewes in our most recent study [5]. Due to the strong influence of the *FecB* mutation on the litter size of sheep, we still showed the AG genotype in Table 2, although the number of individuals was less than ten. We should note that the significant associations between *FecB* and the litter size of UM and DPU only might be considered a preliminary result. Despite this, we still could observe the strong effect of *FecB* on litter size in sheep breeds/populations from MG.

Recently, we discovered novel *GDF9* mutations c.1040T>C (Phe347Ser), g.46544883A>G, g.46547645T>G, and g.46547859C>T linked to the litter size of MG [5,6]. Unfortunately, in this study, none of these variants were connected to UM, DPU, or SFKU. Especially in the missense mutation c.1040T>C (Phe347Ser), the MG litter-size-related C allele was null in the other seven sheep breeds/populations. In addition, the effect of the known c.994A>G (*FecG^A^*) mutation on the litter size of SFKU was observed in this study, despite the small sample size. Although the c.994A>G (*FecG^A^*) variant was discovered in Cambridge and Belclare sheep in Europe [14], the association between this variation and litter size was first demonstrated in Araucana creole sheep in Chile [31]. Therefore, we should note that the current study could not accurately explain how the season, regarding the frequency of the litter-size-associated G allele, was related to higher in SFKU than other sheep breeds/populations; it may be caused by SFK ram. Of course, future studies should confirm this association in a larger sheep population. 

In our recent study, we identified the g.509807863G>A, c.31-33CTTinsdel (*B1*), and c.755T>C in *BMP15* in MG. The A allele of g.509807863G>A and the A allele of c.755T>C in *BMP15* were related to an increased litter size in MG [8]. However, the c.31-33CTTinsdel (*B1*) mutation was linked with the litter size of UM in this study. In contrast, the g.509807863G>A and c.755T>C mutations were not associated with litter size in UM, DPU, and SKFU. Ever since the discovery of *BMP15 B1* (c.31-33CTTinsdel) in Cambridge and Belclare sheep [14], there have been some investigations into this mutation, but the correlations between *B1* and litter size in sheep have not been established until three recently published reports in the composite sheep populations of Xinjiang Cele Black [32], New Zealand [33], and Luzhong Mutton Sheep [34] breeds of China. In addition, the Xinjiang Cele Black [32] and Luzhong Mutton Sheep [34] breeds’ litter size was linked to the missense mutation c.755T>C (Lue252Pro) at the same time. Along with associations between the size of the litter in MG and UM and the c.755T>C and c.31-33CTTinsdel (*B1*) mutations, we speculate that the sheep mutations c.755T>C and c.31-33CTTinsdel (*B1*) could alter the BMP15 protein structure, which affects the conception rate and litter size. However, more research is still needed to confirm this notion. Meanwhile, these association results still need to be confirmed in a different and larger sheep population.

For the newly discovered MG c.240C>T and c.279C>T mutations linked to litter size (8) and the known c.185G>A (*FecD*) mutation linked to litter size in Davisdale sheep (14) of *LEPR*, and for the new g.25929637G>A, g.25929679T>C, g.25929819A>G, and g.25929965A>T mutations associated with litter size in MG (9) and the known c.440C>T and c.823C>T mutations linked to litter size in STH (15) of *B4GALNT2*, unfortunately, a relationship between the above mutations and the litter size of UM, DPU, and SFKU was not built in this study. The ovine prolificacy-related alleles in three mutations of *LEPR* exhibited a remarkably high frequency in each sheep breed/population, especially for the G allele of c.185G>A (*FecD*), indicating that the potential of this mutation as a marker is insufficient. In addition, animals with the TC and GA genotypes of g.25929679T>C and g.25929819A>G showed a trend of a higher litter size than the animals with TT and AA genotypes in SFKU, together with significant associations of the two mutations with the litter size in MG (9); these make g.25929679T>C and g.25929819A>G in *B4GALNT2* a good candidate marker for reproductive traits in sheep. Although this gene’s intron 7 region has the polymorphisms g.25929679T>C and g.25929819A>G (9), the well-known candidate polymorphism g.36938224A>T of the *FecL* mutation in Lacaune sheep is also in this region (33). As there is the possibility for a direct effect of the mutation in intron on the reproductive traits in sheep, it is speculated that the g.25929679T>C and g.25929819A>G may be associated with alternative splicing of the *B4GALNT2* mRNA. To clarify the mechanism of the effect, transcriptional analysis for the intron 7 functions of *B4GALNT2* would be required.

In the meantime, reproduction is a complicated process, and many minor genes, as well as some important genes, have an impact on features such as ovulation rate and litter size [35,36]. Drouilhet et al. [37] reported the combined effect of *FecX^L^* (affecting *BMP15*) and *FecL* (affecting *B4GALNT2*) in Lacaune sheep. Similar to this, *FecB* (which affects *BMPR1B*) and *FecX^G^* (which affects *BMP15*) worked together to affect litter size in STH sheep [21]. These results suggest that the genetically regulated ovulation and reproductive features in the various sheep breeds are controlled by a number of mechanisms. The results of this study have demonstrated a correlation between litter size in UM and the mutations c.746A>G (*FecB*) in *BMPR1B* and c.31 33CTTinsdel (*B1*) in *BMP15*. In conclusion, we suggested that, similar to the Romanov sheep breed, a group of various genes may genetically control the UM breed with a minor effect on each gene [38]. Thus, the findings of this study could be applied in MAS to increase the mean litter sizes in populations of UM, DPU, and SFKU sheep as well as other low-prolificacy breeds. Certainly, association results in this study still need to be confirmed in a different and larger sheep population.

## 5. Conclusions

This study performed an association analysis of the 13 new mutations and 7 known ovine prolificacy-related mutations in *BMPRIB*, *GDF9*, *BMP15*, *LEPR*, and *B4GALNT2* genes with litter size in Ujimqin, the F1 population of Dorper × Ujimqin crossbred, and the F1 population of Suffolk × Ujimqin crossbred populations. Among them, the c.746A>G (*FecB*) mutation in *BMPR1B* had significant effects on the litter size of Ujimqin and the F1 population of Dorper × Ujimqin crossbred sheep populations, the c.994A>G (*FecG^A^*) in *GDF9* was significantly associated with the litter size of the F1 population of Suffolk × Ujimqin crossbred population, and the c.31_33CTTinsdel (*B1*) in *BMP15* was significantly associated with the litter size of the Ujimqin sheep population. The results of this study suggested that the three mutations may be potentially effective genetic markers in MAS to improve litter size in sheep. 

## Figures and Tables

**Table 1:** Information of eight sheep breeds/populations.

Breed	Abbreviation	Number of Ewes	Type
Ujimqin	UM	325 (single lamb 264 + twin lambs 61)	Single birth
Dorper × Ujimqin F1	DPU	304 (single lamb 180 + twin lambs 124)	Single birth
Suffolk × Ujimqin F1	SFKU	66 (single lamb 28 + twin lambs 38)	Single birth
Mongolia ^1^	MG	250	Single birth
Sonid	SN	184	Single birth
Tan	Tan	30	Single birth
Hu	Hu	30	Multiple births
Small-tailed Han	STH	30	Multiple births

^1^ The data on Mongolia sheep was referred to [5,6,7,8,9,10].

**Table 2:** Effects of mutations in *BMPR1B* on litter size in sheep.

Sheep Population	Mutation	Genotype	Number	Litter Size
Ujimqin	c.746A>G (Gln249Arg)	AA	320	1.18 ± 0.02 ^a^
		AG	5	1.60 ± 0.24 ^b^
	c.1470G>T (490Thr)	AA	45	1.20 ± 0.05
		AC	155	1.17 ± 0.03
		CC	116	1.21 ± 0.04
Dorper × Ujimqin F1	c.746A>G (Gln249Arg)	AA	300	1.40 ± 0.03 ^a^
		AG	4	2.00 ± 0.00 ^b^
	c.1470G>T (490Thr)	AA	12	1.25 ± 0.13
		AC	111	1.41± 0.05
		CC	181	1.42 ± 0.04
Suffolk × Ujimqin F1	c.1470G>T (490Thr)	AA	14	1.50 ± 0.14
		AC	40	1.63 ± 0.08
		CC	12	1.28 ± 0.06

Note: ^a, b^: *p* < 0.05.

**Table 3:** Effects of mutations in *GDF9* on litter size in sheep.

Breed	Mutation	Genotype	Number	Litter Size
Ujimqin	g.46547859C>T	CC	244	1.19 ± 0.03
		CT	79	1.18 ± 0.04
	g.46547645T>G	TT	198	1.20 ± 0.03
		TG	118	1.16 ± 0.03
	c.260G>A (Arg87His)	GG	292	1.19 ± 0.02
		GA	32	1.16 ± 0.07
	c.994A>G (Val322Ile)	AA	299	1.19 ± 0.02
		AG	24	1.21 ± 0.08
	g.46544883A>G	AA	244	1.19 ± 0.03
		AG	79	1.18 ± 0.04
Dorper × Ujimqin F1	g.46547859C>T	CC	189	1.40 ± 0.04
		CT	107	1.43 ± 0.05
	g.46547645T>G	TT	178	1.40 ± 0.04
		GT	113	1.42 ± 0.05
	c.994A>G (Val322Ile)	AA	279	1.41 ± 0.03
		AG	28	1.43 ± 0.09
	g.46544883A>G	AA	189	1.40 ± 0.04
		AG	106	1.43 ± 0.05
Suffolk × Ujimqin F1	g.46547859C>T	CC	51	1.59 ± 0.07
		CT	13	1.61 ± 0.14
	g.46547645T>G	TT	48	1.60 ± 0.07
		TG	16	1.56 ± 0.13
	c.994A>G (Val322Ile)	AA	46	1.48 ± 0.07 ^a^
		AG	20	1.80 ± 0.09 ^b^
	g.46544883A>G	AA	51	1.59 ± 0.07
		AG	13	1.62 ± 0.14

Note: ^a, b^: *p* < 0.05.

**Table 4:** Effects of mutations in *BMP15* on litter size in sheep.

Breed	Mutation	Genotype	Number	Litter Size
Ujimqin	g.509807863G>A	GG	287	1.19 ± 0.02
		GA	38	1.18 ± 0.06
	c.31_33CTTinsdel (11Leu deletion)	CCT.CCT	191	1.13 ± 0.02 ^A^
		CCT.DEL	112	1.27 ± 0.04 ^B^
		DEL.DEL	22	1.27 ± 0.10
	c.755T>C (Lue252Pro)	TT	259	1.19 ± 0.02
		TC	64	1.20 ± 0.05
Dorper × Ujimqin F1	c.31_33CTTinsdel (11Leu deletion)	CCT.CCT	84	1.38 ± 0.05
		CCT.DEL	167	1.41 ± 0.04
		DEL.DEL	53	1.43 ± 0.07
	c.755T>C (Lue252Pro)	TT	235	1.41 ± 0.03
		TC	66	1.41 ± 0.06
Suffolk × Ujimqin F1	c.31_33CTTinsdel (11Leu_deletion)	CCT.CCT	44	1.59 ± 0.07
		CCT.DEL	20	1.60 ± 0.11

Note: ^A, B^: *p* < 0.01.

**Table 5:** Effects of mutations in *LEPR* on litter size in sheep.

Breed	Mutation	Genotype	Number	Litter Size
Ujimqin	c.185G>A (Arg62His)	GG	249	1.21 ± 0.03
		GA	71	1.11 ± 0.04
	c.240C>T (80Asn)	CC	60	1.10 ± 0.04
		CT	147	1.18 ± 0.03
		TT	118	1.24 ± 0.04
	c.279C>T (93Ser)	CC	60	1.10 ± 0.04
		CT	147	1.18 ± 0.03
		TT	118	1.24 ± 0.04
Dorper × Ujimqin F1	c.185G>A (Arg62His)	GG	249	1.41 ± 0.03
		GA	55	1.40 ± 0.07
	c.240C>T (80Asn)	CC	16	1.38 ± 0.13
		CT	121	1.43 ± 0.06
		TT	167	1.40 ± 0.04
	c.279C>T (93Ser)	CC	16	1.38 ± 0.13
		CT	121	1.43 ± 0.06
		TT	167	1.40 ± 0.04
Suffolk × Ujimqin F1	c.185G>A (Arg62His)	GG	35	1.51 ± 0.09
		GA	25	1.56 ± 0.10
	c.240C>T (80Asn)	CT	35	1.66 ± 0.08
		TT	26	1.46 ± 0.10
	c.279C>T (93Ser)	CT	35	1.66 ± 0.08
		TT	26	1.46 ± 0.10

**Table 6:** Effects of mutations in *B4GALNT2* on litter size in sheep.

Breed	Mutation	Genotype	Number	Litter Size
Ujimqin	c.440C>T (Pro160Leu)	CC	255	1.18 ± 0.02
		CT	66	1.20 ± 0.05
	g.25929637G>A	GG	289	1.19 ± 0.02
		GA	35	1.17 ± 0.06
	g.25929679T>C	TT	229	1.18 ± 0.03
		TC	93	1.18 ± 0.04
	g.25929819A>G	AA	229	1.18 ± 0.03
		AG	93	1.18 ± 0.04
	g.25929965A>T	AA	289	1.19 ± 0.02
		AT	33	1.12 ± 0.06
	c.823C>T (Pro288Ser)	CC	218	1.22 ± 0.03
		CT	93	1.14 ± 0.04
		TT	14	1.07 ± 0.07
Dorper × Ujimqin F1	c.440C>T (Pro160Leu)	CC	251	1.43 ± 0.03
		CT	52	1.33 ± 0.07
	g.25929679T>C	TT	243	1.39 ± 0.03
		TC	59	1.44 ± 0.07
	g.25929819A>G	AA	243	1.40 ± 0.03
		AG	59	1.44 ± 0.07
	c.823C>T (Pro288Ser)	CC	244	1.39 ± 0.03
		CT	57	1.49 ± 0.07
Suffolk × Ujimqin F1	c.440C>T (Pro160Leu)	CC	51	1.59 ± 0.07
		CT	14	1.57 ± 0.14
	g.25929679T>C	TT	20	1.40 ± 0.11
		TC	39	1.64 ± 0.08
	g.25929819A>G	AA	20	1.40 ± 0.11
		AG	39	1.64 ± 0.08

## Data Availability

Not applicable.

## Supplementary Materials

The following supporting information can be downloaded at: https://www.mdpi.com/article/10.3390/vetsci10040258/s1, Table S1: Genotypic, allelic frequencies and diversity parameters of two mutations of *BMPR1B* in eight sheep populations; Table S2: Genotypic, allelic frequencies, and diversity parameters of six mutations of *GDF9* in eight sheep populations; Table S3: Genotypic, allelic frequencies, and diversity parameters of three mutations of *BMP15* in eight sheep populations; Table S4: Genotypic, allelic frequencies, and diversity parameters of three mutations of *LEPR* in eight sheep populations; Table S5: Genotypic, allelic frequencies, and diversity parameters of six mutations of *B4GALNT2* in eight sheep populations; Table S6: Statistical significance for differences in the allele frequency of *BMPR1B* variants among eight sheep populations; Table S7: Statistical significance for differences in the allele frequency of *GDF9* variants among eight sheep populations; Table S8: Statistical significance for differences in the allele frequency of *BMP15* variants among eight sheep populations; Table S9: Statistical significance for differences in the allele frequency of *LEPR* variants among eight sheep populations; Table S10: Statistical significance for differences in the allele frequency of *B4GALNT2* variants among eight sheep populations.
