# Supplementary material for: Effects of New Mutations in BMPRIB, GDF9, BMP15, LEPR, and B4GALNT2 Genes on Litter Size in Sheep
Source: Vet Sci. 2023 Mar 28;10(4):258. doi: 10.3390/vetsci10040258 (PMC10141719; doi:10.3390/vetsci10040258)
Supplement: Supplementary file 1 [file vetsci-10-00258-s001.zip › vetsci-2219749-supplementary/Supplementary Materials Table S1-S10/Table S1. two mutations of BMPR1B in eight sheep populations..pdf]

Table S1. Genotypic, allelic frequencies and diversity parameters of two mutations of *BMPR1B* in eight sheep populations.

**Locus/Breed**	**Genotype frequency**			**Allele frequency**		**Diversity parameter**				
						**Ho**	**He**	**n_e**	**PIC**	**$\chi 2$ (HWE)**
c.746A>G (Gln249Arg)	GG	AG	AA	G	A					
UM	0.985	0.015	0.000	0.992	0.008	0.985	0.015	1.016	0.016	0.020
DBU	0.987	0.013	0.000	0.993	0.007	0.987	0.013	1.013	0.014	0.013
SFKU	1.000	0.000	0.000	1.000	0.000	1.000	0.000	1.000	0.000	-
SN	1.000	0.000	0.000	1.000	0.000	1.000	0.000	1.000	0.000	-
MG	0.962	0.038	0.000	0.981	0.019	0.962	0.038	1.039	0.037	0.100
Tan	0.767	0.200	0.033	0.867	0.133	0.769	0.231	1.301	0.204	0.544
Hu	0.167	0.333	0.500	0.333	0.667	0.556	0.444	1.800	0.346	1.875
STH	0.000	0.667	0.333	0.333	0.667	0.556	0.444	1.800	0.346	7.500
c.1470G>T (490Thr)	AA	AC	CC	A	C					
UM	0.166	0.477	0.357	0.405	0.595	0.518	0.482	1.930	0.366	0.033
DBU	0.039	0.365	0.595	0.222	0.778	0.655	0.345	1.528	0.286	0.984
SFKU	0.212	0.606	0.182	0.515	0.485	0.500	0.500	1.998	0.375	3.001
SNT	0.147	0.505	0.348	0.399	0.601	0.520	0.480	1.922	0.365	0.526
MG	0.160	0.412	0.428	0.366	0.634	0.536	0.464	1.866	0.356	3.149
Tan	0.233	0.533	0.233	0.500	0.500	0.500	0.500	2.000	0.375	0.133
Hu	0.000	0.033	0.967	0.017	0.983	0.967	0.033	1.034	0.033	0.009
STH	0.000	0.333	0.667	0.167	0.833	0.722	0.278	1.385	0.240	1.200

*UM: Ujimqin; DPU: Dorper × Ujimqin; SFKU: Suffolk × Ujimqin; SN: Sonid; MG: Mongolia; STH: Small-tailed Han; H_o: observed heterozygosity; H_e: expected heterozygosity; n_e: effective allele numbers; *PIC*: polymorphism information content; *HWE*: Hardy-Weinberg equilibrium; [1] The classification was conducted according to the PIC values (PIC value < 0.25, low polymorphism; 0.25 < PIC value < 0.5, moderate polymorphism; and PIC value > 0.5, high polymorphism); [2] No Hardy-Weinberg departure was detected from the obtained genotype frequencies.
