# Supplementary material for: Effects of New Mutations in BMPRIB, GDF9, BMP15, LEPR, and B4GALNT2 Genes on Litter Size in Sheep
Source: Vet Sci. 2023 Mar 28;10(4):258. doi: 10.3390/vetsci10040258 (PMC10141719; doi:10.3390/vetsci10040258)
Supplement: Supplementary file 1 [file vetsci-10-00258-s001.zip › vetsci-2219749-supplementary/Supplementary Materials Table S1-S10/Table S2. six mutations of GDF9 in eight sheep populations..pdf]

**Table S2**. Genotypic, allelic frequencies and diversity parameters of six mutations of *GDF9* in eight sheep populations.

| Locus/Breed | Genotype frequency | | | Allele frequency | | Diversity parameter | | | | |
|---|---|---|---|---|---|---|---|---|---|---|
| | | | | | | **Ho** | **He** | $n_e$ | **PIC** | $\chi 2$ **(HWE)** |
| g.46547859C>T | CC | CT | TT | C | T | | | | | |
| UM | 0.751 | 0.243 | 0.006 | 0.872 | 0.128 | 0.777 | 0.223 | 1.287 | 0.198 | 2.699 |
| DBU | 0.622 | 0.352 | 0.026 | 0.798 | 0.202 | 0.677 | 0.323 | 1.477 | 0.270 | 2.492 |
| SFKU | 0.773 | 0.197 | 0.030 | 0.871 | 0.129 | 0.776 | 0.224 | 1.289 | 0.199 | 0.986 |
| SN | 0.770 | 0.202 | 0.027 | 0.872 | 0.128 | 0.776 | 0.224 | 1.288 | 0.198 | 1.714 |
| MG | 0.816 | 0.176 | 0.008 | 0.904 | 0.096 | 0.826 | 0.174 | 1.210 | 0.159 | 0.049 |
| Tan | 0.800 | 0.200 | 0.000 | 0.900 | 0.100 | 0.820 | 0.180 | 1.220 | 0.164 | 0.370 |
| Hu | 0.767 | 0.200 | 0.033 | 0.867 | 0.133 | 0.769 | 0.231 | 1.301 | 0.204 | 0.544 |
| STH | 0.733 | 0.233 | 0.033 | 0.850 | 0.150 | 0.745 | 0.255 | 1.342 | 0.222 | 0.217 |
| g.46547645T>G | TT | TG | GG | T | G | | | | | |
| UM | 0.609 | 0.363 | 0.028 | 0.791 | 0.209 | 0.669 | 0.331 | 1.495 | 0.276 | 3.072 |
| DBU | 0.586 | 0.372 | 0.043 | 0.771 | 0.229 | 0.647 | 0.353 | 1.545 | 0.291 | 0.883 |
| SFKU | 0.727 | 0.242 | 0.030 | 0.848 | 0.152 | 0.743 | 0.257 | 1.346 | 0.225 | 0.216 |
| SN | 0.685 | 0.272 | 0.043 | 0.821 | 0.179 | 0.706 | 0.294 | 1.417 | 0.251 | 1.087 |
| MG | 0.712 | 0.258 | 0.031 | 0.840 | 0.160 | 0.732 | 0.268 | 1.367 | 0.233 | 260.000 |
| Tan | 0.700 | 0.300 | 0.000 | 0.850 | 0.150 | 0.745 | 0.255 | 1.342 | 0.222 | 0.934 |
| Hu | 0.667 | 0.300 | 0.033 | 0.817 | 0.183 | 0.701 | 0.299 | 1.427 | 0.254 | 0.000 |
| STH | 0.633 | 0.333 | 0.033 | 0.800 | 0.200 | 0.680 | 0.320 | 1.471 | 0.269 | 0.052 |
| c.260G>A (Arg87His) | AA | AG | GG | A | G | | | | | |
| UM | 0.898 | 0.098 | 0.003 | 0.948 | 0.052 | 0.901 | 0.099 | 1.110 | 0.094 | 0.015 |
| DBU | 0.974 | 0.026 | 0.000 | 0.987 | 0.013 | 0.974 | 0.026 | 1.027 | 0.025 | 0.054 |
| SFKU | 0.955 | 0.045 | 0.000 | 0.977 | 0.023 | 0.956 | 0.044 | 1.046 | 0.044 | 0.036 |
| SN | 0.923 | 0.077 | 0.000 | 0.962 | 0.038 | 0.926 | 0.074 | 1.079 | 0.070 | 0.289 |

| | | | | | | | | | | |
|---|---|---|---|---|---|---|---|---|---|---|
| MG | 0.892 | 0.104 | 0.004 | 0.944 | 0.056 | 0.859 | 0.105 | 1.118 | 0.100 | 0.051 |
| Tan | 0.967 | 0.033 | 0.000 | 0.983 | 0.017 | 0.967 | 0.033 | 1.034 | 0.033 | 0.009 |
| Hu | 0.967 | 0.033 | 0.000 | 0.983 | 0.017 | 0.967 | 0.033 | 1.034 | 0.033 | 0.009 |
| STH | 0.967 | 0.033 | 0.000 | 0.983 | 0.017 | 0.967 | 0.033 | 1.034 | 0.033 | 0.009 |
| c.994A>G (Val322Ile) | AA | AG | GG | A | G | | | | | |
| UM | 0.920 | 0.074 | 0.006 | 0.957 | 0.043 | 0.918 | 0.082 | 1.090 | 0.079 | 3.534 |
| DBU | 0.908 | 0.092 | 0.000 | 0.954 | 0.046 | 0.912 | 0.088 | 1.096 | 0.084 | 0.708 |
| SFKU | 0.697 | 0.303 | 0.000 | 0.848 | 0.152 | 0.743 | 0.257 | 1.346 | 0.225 | 2.105 |
| SN | 0.935 | 0.065 | 0.000 | 0.967 | 0.033 | 0.937 | 0.063 | 1.067 | 0.062 | 0.209 |
| MG | 0.854 | 0.138 | 0.008 | 0.923 | 0.077 | 0.858 | 0.142 | 1.166 | 0.132 | 0.163 |
| Tan | 1.000 | 0.000 | 0.000 | 1.000 | 0.000 | 1.000 | 0.000 | 1.000 | 0.000 | - |
| Hu | 1.000 | 0.000 | 0.000 | 1.000 | 0.000 | 1.000 | 0.000 | 1.000 | 0.000 | - |
| STH | 0.967 | 0.033 | 0.000 | 0.983 | 0.017 | 0.967 | 0.033 | 1.034 | 0.033 | 0.009 |
| c.1040T>C (Phe347Ser) | TT | TC | CC | T | C | | | | | |
| UM | 1.000 | 0.000 | 0.000 | 1.000 | 0.000 | 1.000 | 0.000 | 1.000 | 0.000 | - |
| DBU | 0.997 | 0.003 | 0.000 | 0.998 | 0.002 | 0.997 | 0.003 | 1.003 | 0.004 | 0.001 |
| SFKU | 1.000 | 0.000 | 0.000 | 1.000 | 0.000 | 1.000 | 0.000 | 1.000 | 0.000 | - |
| SN | 1.000 | 0.000 | 0.000 | 1.000 | 0.000 | 1.000 | 0.000 | 1.000 | 0.000 | - |
| MG | 0.932 | 0.068 | 0.000 | 0.966 | 0.034 | 0.934 | 0.066 | 1.070 | 0.064 | 250.000 |
| Tan | 1.000 | 0.000 | 0.000 | 1.000 | 0.000 | 1.000 | 0.000 | 1.000 | 0.000 | - |
| Hu | 1.000 | 0.000 | 0.000 | 1.000 | 0.000 | 1.000 | 0.000 | 1.000 | 0.000 | - |
| STH | 1.000 | 0.000 | 0.000 | 1.000 | 0.000 | 1.000 | 0.000 | 1.000 | 0.000 | - |
| g.46544883A>G | GG | GA | AA | G | A | | | | | |
| UM | 0.751 | 0.243 | 0.006 | 0.872 | 0.128 | 0.777 | 0.223 | 1.287 | 0.198 | 2.699 |
| DBU | 0.622 | 0.349 | 0.030 | 0.796 | 0.204 | 0.675 | 0.325 | 1.481 | 0.272 | 1.658 |
| SFKU | 0.773 | 0.197 | 0.030 | 0.871 | 0.129 | 0.776 | 0.224 | 1.289 | 0.199 | 0.986 |

| | | | | | | | | | | |
|---|---|---|---|---|---|---|---|---|---|---|
| SN | 0.764 | 0.209 | 0.027 | 0.868 | 0.132 | 0.771 | 0.229 | 1.297 | 0.203 | 1.412 |
| MG | 0.764 | 0.228 | 0.008 | 0.878 | 0.122 | 0.786 | 0.214 | 1.273 | 0.191 | 1.033 |
| Tan | 0.800 | 0.200 | 0.000 | 0.900 | 0.100 | 0.820 | 0.180 | 1.220 | 0.164 | 0.370 |
| Hu | 0.767 | 0.200 | 0.033 | 0.867 | 0.133 | 0.769 | 0.231 | 1.301 | 0.204 | 0.544 |
| STH | 0.733 | 0.233 | 0.033 | 0.850 | 0.150 | 0.745 | 0.255 | 1.342 | 0.222 | 0.217 |

*UM: Ujimqin; DPU: Dorper × Ujimqin; SFKU: Suffolk × Ujimqin; SN: Sonid; MG: Mongolia; STH: Small-tailed Han; $H_o$: observed heterozygosity; $H_e$: expected heterozygosity; $n_e$: effective allele numbers; *PIC*: polymorphism information content; *HWE*: Hardy-Weinberg equilibrium; [1] The classification was conducted according to the PIC values (PIC value < 0.25, low polymorphism; 0.25 < PIC value < 0.5, moderate polymorphism; and PIC value > 0.5, high polymorphism); [2] No Hardy-Weinberg departure was detected from the obtained genotype frequencies.
