# Supplementary material for: Effects of New Mutations in BMPRIB, GDF9, BMP15, LEPR, and B4GALNT2 Genes on Litter Size in Sheep
Source: Vet Sci. 2023 Mar 28;10(4):258. doi: 10.3390/vetsci10040258 (PMC10141719; doi:10.3390/vetsci10040258)
Supplement: Supplementary file 1 [file vetsci-10-00258-s001.zip › vetsci-2219749-supplementary/Supplementary Materials Table S1-S10/Table S3. three mutations of BMP15 in eight sheep populations..pdf]

Table S3. Genotypic, allelic frequencies and diversity parameters of three mutations of *BMP15* in eight sheep populations.

Locus/Breed	Genotype frequency			Allele frequency		Diversity parameter				
						Ho	He	n_e	PIC	χ2 (HWE)
g.509807863G>A	GG	GA	AA	G	A					
UM	0.883	0.117	0.000	0.942	0.058	0.890	0.110	1.124	0.103	1.253
DBU	0.977	0.023	0.000	0.988	0.012	0.977	0.023	1.023	0.023	0.041
SFKU	0.985	0.015	0.000	0.992	0.008	0.985	0.015	1.015	0.016	0.004
SN	0.995	0.005	0.000	0.997	0.003	0.995	0.005	1.005	0.006	0.001
MG	0.984	0.016	0.000	0.992	0.008	0.984	0.016	1.016	0.016	0.016
Tan	1.000	0.000	0.000	1.000	0.000	1.000	0.000	1.000	0.000	-
Hu	1.000	0.000	0.000	1.000	0.000	1.000	0.000	1.000	0.000	-
STH	1.000	0.000	0.000	1.000	0.000	1.000	0.000	1.000	0.000	-
c.31_33CTTinsdel (11Leu deletion)	CTT	CTT.DEL	DEL	CTT	DEL					
UM	0.588	0.345	0.068	0.760	0.240	0.635	0.365	1.574	0.298	0.995
DBU	0.276	0.549	0.174	0.551	0.449	0.505	0.495	1.979	0.372	3.694
SFKU	0.667	0.303	0.030	0.818	0.182	0.702	0.298	1.424	0.253	0.023
SN	0.516	0.397	0.087	0.715	0.285	0.592	0.408	1.689	0.325	0.136
MG	0.628	0.348	0.024	0.802	0.198	0.682	0.318	1.465	0.267	2.292
Tan	0.567	0.367	0.067	0.750	0.250	0.625	0.375	1.600	0.305	0.015
Hu	0.633	0.300	0.067	0.783	0.217	0.661	0.339	1.514	0.282	0.405
STH	0.467	0.433	0.100	0.683	0.317	0.567	0.433	1.763	0.339	0.000
c.755T>C (Lue252Pro)	TT	TC	CC	T	C					
UM	0.797	0.197	0.006	0.895	0.105	0.813	0.187	1.231	0.170	0.850
DBU	0.773	0.217	0.010	0.882	0.118	0.791	0.209	1.264	0.186	0.482
SFKU	0.879	0.121	0.000	0.939	0.061	0.886	0.114	1.128	0.108	0.275
SN	0.821	0.143	0.036	0.893	0.107	0.809	0.191	1.237	0.173	10.782

MG	0.848	0.144	0.008	0.920	0.080	0.853	0.147	1.173	0.136	250.000
Tan	0.964	0.036	0.000	0.982	0.018	0.965	0.035	1.036	0.035	0.009
Hu	0.700	0.300	0.000	0.850	0.150	0.745	0.255	1.342	0.222	0.934
STH	0.621	0.379	0.000	0.810	0.190	0.693	0.307	1.444	0.260	1.589

*UM: Ujimqin; DPU: Dorper × Ujimqin; SFKU: Suffolk × Ujimqin; SN: Sonid; MG: Mongolia; STH: Small-tailed Han; H_o: observed heterozygosity; H_e: expected heterozygosity; n_e: effective allele numbers; *PIC*: polymorphism information content; *HWE*: Hardy-Weinberg equilibrium; [1] The classification was conducted according to the PIC values (PIC value < 0.25, low polymorphism; 0.25 < PIC value < 0.5, moderate polymorphism; and PIC value > 0.5, high polymorphism); [2] No Hardy-Weinberg departure was detected from the obtained genotype frequencies.
