# Supplementary material for: Effects of New Mutations in BMPRIB, GDF9, BMP15, LEPR, and B4GALNT2 Genes on Litter Size in Sheep
Source: Vet Sci. 2023 Mar 28;10(4):258. doi: 10.3390/vetsci10040258 (PMC10141719; doi:10.3390/vetsci10040258)
Supplement: Supplementary file 1 [file vetsci-10-00258-s001.zip › vetsci-2219749-supplementary/Supplementary Materials Table S1-S10/Table S4. three mutations of LEPR in eight sheep populations..pdf]

Table S4. Genotypic, allelic frequencies and diversity parameters of three mutations of *LEPR* in eight sheep populations.

Locus/Breed	Genotype frequency			Allele frequency		Diversity parameter				
						Ho	He	n_e	PIC	χ2 (HWE)
c.185G>A (Arg62His)	GG	GA	AA	G	A					
UM	0.766	0.218	0.015	0.875	0.125	0.782	0.218	1.279	0.195	0.001
DBU	0.819	0.181	0.000	0.910	0.090	0.835	0.165	1.197	0.150	3.007
SFKU	0.530	0.379	0.091	0.720	0.280	0.597	0.403	1.676	0.322	0.247
SN	0.661	0.311	0.028	0.816	0.184	0.700	0.300	1.428	0.255	0.235
MG	0.972	0.000	0.028	0.972	0.028	0.946	0.054	1.058	0.053	250.000
Tan	0.833	0.167	0.000	0.917	0.083	0.847	0.153	1.180	0.141	0.248
Hu	0.667	0.300	0.033	0.817	0.183	0.701	0.299	1.427	0.254	0.000
STH	0.767	0.200	0.033	0.867	0.133	0.769	0.231	1.301	0.204	0.544
c.240C>T (80Asn)	CC	CT	TT	C	T					
UM	0.185	0.452	0.363	0.411	0.589	0.516	0.484	1.938	0.367	1.400
DBU	0.053	0.398	0.549	0.252	0.748	0.623	0.377	1.604	0.306	0.980
SFKU	0.076	0.530	0.394	0.341	0.659	0.551	0.449	1.816	0.348	2.140
SN	0.120	0.495	0.386	0.367	0.633	0.535	0.465	1.868	0.357	0.769
MG	0.128	0.460	0.412	0.358	0.642	0.540	0.460	1.851	0.354	0.000
Tan	0.267	0.567	0.167	0.550	0.450	0.505	0.495	1.980	0.372	0.629
Hu	0.200	0.500	0.300	0.450	0.550	0.505	0.495	1.980	0.372	0.003
STH	0.300	0.400	0.300	0.500	0.500	0.500	0.500	2.000	0.375	1.200
c.279C>T (93Ser)	CC	CT	TT	C	T					
UM	0.185	0.452	0.363	0.411	0.589	0.516	0.484	1.938	0.367	1.400
DBU	0.053	0.398	0.549	0.252	0.748	0.623	0.377	1.604	0.306	0.980
SFKU	0.076	0.530	0.394	0.341	0.659	0.551	0.449	1.816	0.348	2.140
SN	0.121	0.468	0.410	0.355	0.645	0.542	0.458	1.846	0.353	0.082

MG	0.128	0.460	0.412	0.358	0.642	0.540	0.460	1.851	0.354	0.000
Tan	0.267	0.567	0.167	0.550	0.450	0.505	0.495	1.980	0.372	0.629
Hu	0.200	0.500	0.300	0.450	0.550	0.505	0.495	1.980	0.372	0.003
STH	0.300	0.400	0.300	0.500	0.500	0.500	0.500	2.000	0.375	1.200

*UM: Ujimqin; DPU: Dorper × Ujimqin; SFKU: Suffolk × Ujimqin; SN: Sonid; MG: Mongolia; STH: Small-tailed Han; H_o: observed heterozygosity; H_e: expected heterozygosity; n_e: effective allele numbers; *PIC*: polymorphism information content; *HWE*: Hardy-Weinberg equilibrium; [1] The classification was conducted according to the PIC values (PIC value < 0.25, low polymorphism; 0.25 < PIC value < 0.5, moderate polymorphism; and PIC value > 0.5, high polymorphism); [2] No Hardy-Weinberg departure was detected from the obtained genotype frequencies.
