# Supplementary material for: Effects of New Mutations in BMPRIB, GDF9, BMP15, LEPR, and B4GALNT2 Genes on Litter Size in Sheep
Source: Vet Sci. 2023 Mar 28;10(4):258. doi: 10.3390/vetsci10040258 (PMC10141719; doi:10.3390/vetsci10040258)
Supplement: Supplementary file 1 [file vetsci-10-00258-s001.zip › vetsci-2219749-supplementary/Supplementary Materials Table S1-S10/Table S5. six mutations of B4GALNT2 in eight sheep populations..pdf]

Table S5. Genotypic, allelic frequencies and diversity parameters of six mutations of *B4GALNT2* in eight sheep populations.

Locus/Breed	Genotype frequency			Allele frequency		Diversity parameter				
						Ho	He	n_e	PIC	$\chi 2$ (HWE)
c.440 C>T (Pro160Leu)	CC	CT	TT	C	T					
UM	0.785	0.203	0.012	0.886	0.114	0.798	0.202	1.253	0.182	0.014
DBU	0.826	0.171	0.003	0.911	0.089	0.838	0.162	1.193	0.149	0.982
SFKU	0.773	0.212	0.015	0.879	0.121	0.787	0.213	1.271	0.190	0.001
SN	0.810	0.179	0.011	0.899	0.101	0.819	0.181	1.221	0.165	0.013
MG	0.760	0.212	0.028	0.866	0.134	0.768	0.232	1.302	0.205	1.873
Tan	0.967	0.000	0.033	0.967	0.033	0.936	0.064	1.069	0.062	30.000
Hu	0.567	0.300	0.133	0.717	0.283	0.594	0.406	1.684	0.323	2.048
STH	0.733	0.233	0.033	0.850	0.150	0.745	0.255	1.342	0.222	0.217
g.25929637G>A	GG	GA	AA	G	A					
UM	0.889	0.108	0.003	0.943	0.057	0.893	0.107	1.120	0.102	0.003
DBU	0.970	0.030	0.000	0.985	0.015	0.971	0.029	1.030	0.029	0.069
SFKU	0.985	0.015	0.000	0.992	0.008	0.985	0.015	1.015	0.016	0.004
SN	0.808	0.175	0.017	0.895	0.105	0.813	0.187	1.230	0.170	0.733
MG	0.828	0.156	0.016	0.906	0.094	0.830	0.170	1.205	0.156	1.769
Tan	0.821	0.179	0.000	0.911	0.089	0.837	0.163	1.194	0.149	0.269
Hu	0.967	0.033	0.000	0.983	0.017	0.967	0.033	1.034	0.033	0.009
STH	0.897	0.103	0.000	0.948	0.052	0.902	0.098	1.109	0.094	0.086
g.25929679T>C	TT	TC	CC	T	C					
UM	0.705	0.286	0.009	0.848	0.152	0.742	0.258	1.348	0.225	3.803
DBU	0.799	0.194	0.007	0.896	0.104	0.814	0.186	1.228	0.169	0.609
SFKU	0.303	0.591	0.106	0.598	0.402	0.519	0.481	1.925	0.365	3.477
SN	0.692	0.217	0.091	0.801	0.199	0.681	0.319	1.469	0.268	14.714

MG	0.640	0.324	0.036	0.802	0.198	0.682	0.318	1.465	0.267	250.000
Tan	0.750	0.179	0.071	0.839	0.161	0.730	0.270	1.369	0.234	3.200
Hu	0.967	0.033	0.000	0.983	0.017	0.967	0.033	1.034	0.033	0.009
STH	0.840	0.120	0.040	0.900	0.100	0.820	0.180	1.220	0.164	2.778
g.25929819A>G	GG	GA	AA	G	A					
UM	0.705	0.286	0.009	0.848	0.152	0.742	0.258	1.348	0.225	3.803
DBU	0.799	0.194	0.007	0.896	0.104	0.814	0.186	1.228	0.169	0.609
SFKU	0.303	0.591	0.106	0.598	0.402	0.519	0.481	1.925	0.365	3.477
SN	0.549	0.380	0.071	0.739	0.261	0.614	0.386	1.628	0.311	0.033
MG	0.648	0.328	0.024	0.812	0.188	0.695	0.305	1.439	0.259	1.381
Tan	0.700	0.233	0.067	0.817	0.183	0.701	0.299	1.427	0.254	1.462
Hu	0.967	0.033	0.000	0.983	0.017	0.967	0.033	1.034	0.033	0.009
STH	0.700	0.267	0.033	0.833	0.167	0.722	0.278	1.385	0.240	0.048
g.25929965A>T	AA	AT	TT	A	T					
UM	0.889	0.102	0.009	0.940	0.060	0.887	0.113	1.127	0.106	3.239
DBU	0.970	0.030	0.000	0.985	0.015	0.971	0.029	1.030	0.029	0.069
SFKU	0.985	0.000	0.015	0.985	0.015	0.970	0.030	1.031	0.029	66.000
SN	0.777	0.158	0.065	0.856	0.144	0.753	0.247	1.327	0.216	23.948
MG	0.828	0.156	0.016	0.906	0.094	0.830	0.170	1.205	0.156	1.769
Tan	0.767	0.167	0.067	0.850	0.150	0.745	0.255	1.342	0.222	3.600
Hu	0.967	0.033	0.000	0.983	0.017	0.967	0.033	1.034	0.033	0.009
STH	0.867	0.100	0.033	0.917	0.083	0.847	0.153	1.180	0.141	3.580
c.823C>T (Pro288Ser)	CC	CT	TT	C	T					
UM	0.671	0.286	0.043	0.814	0.186	0.697	0.303	1.435	0.257	1.005
DBU	0.803	0.188	0.010	0.896	0.104	0.814	0.186	1.228	0.169	0.027
SFKU	0.909	0.091	0.000	0.955	0.045	0.913	0.087	1.095	0.082	0.150

SN	0.703	0.258	0.038	0.832	0.168	0.721	0.279	1.387	0.240	1.007
MG	0.656	0.292	0.052	0.802	0.198	0.682	0.318	1.465	0.267	1.623
Tan	0.933	0.067	0.000	0.967	0.033	0.936	0.064	1.069	0.062	0.036
Hu	0.600	0.367	0.033	0.783	0.217	0.661	0.339	1.514	0.282	0.193
STH	0.828	0.138	0.034	0.897	0.103	0.815	0.185	1.228	0.168	1.907

*UM: Ujimqin; DPU: Dorper × Ujimqin; SFKU: Suffolk × Ujimqin; SN: Sonid; MG: Mongolia; STH: Small-tailed Han; H_o: observed heterozygosity; H_e: expected heterozygosity; n_e: effective allele numbers; *PIC*: polymorphism information content; *HWE*: Hardy-Weinberg equilibrium; [1] The classification was conducted according to the PIC values (PIC value < 0.25, low polymorphism; 0.25 < PIC value < 0.5, moderate polymorphism; and PIC value > 0.5, high polymorphism); [2] No Hardy-Weinberg departure was detected from the obtained genotype frequencies.
