# Supplementary material for: Effects of New Mutations in BMPRIB, GDF9, BMP15, LEPR, and B4GALNT2 Genes on Litter Size in Sheep
Source: Vet Sci. 2023 Mar 28;10(4):258. doi: 10.3390/vetsci10040258 (PMC10141719; doi:10.3390/vetsci10040258)
Supplement: Supplementary file 1 [file vetsci-10-00258-s001.zip › vetsci-2219749-supplementary/Supplementary Materials Table S1-S10/Table S7. GDF9 variant among eight sheep populations..pdf]

Table S7. Statistical significance for differences in the allele frequency of *GDF9* variant among eight sheep populations.

Variant/Breed	DPU	SFKU	SN	MG	Tan	Hu	STH
g.46547859C>T							
UM	***	n.s.	n.s.	n.s.	n.s.	n.s.	n.s.
DPU		n.s.	**	***	n.s.	n.s.	n.s.
SFKU			n.s.	n.s.	n.s.	n.s.	n.s.
SN				n.s.	n.s.	n.s.	n.s.
MG					n.s.	n.s.	n.s.
Tan						n.s.	n.s.
Hu							n.s.
g.46547645T>G							
UM	n.s.	n.s.	n.s.	—	n.s.	n.s.	n.s.
DPU		n.s.	n.s.	**	n.s.	n.s.	n.s.
SFKU			n.s.	n.s.	n.s.	n.s.	n.s.
SN				n.s.	n.s.	n.s.	n.s.
MG					n.s.	n.s.	n.s.
Tan						n.s.	n.s.
Hu							n.s.
c.260G>A (Arg87His)							
UM	***	n.s.	n.s.	***	n.s.	n.s.	n.s.
DPU		n.s.	*	***	n.s.	n.s.	n.s.
SFKU			n.s.	***	n.s.	n.s.	n.s.
SN				**	n.s.	n.s.	n.s.
MG					***	***	***
Tan						n.s.	n.s.
Hu							n.s.
c.994A>G (Val322Ile)							
UM	n.s.	***	n.s.	***	n.s.	n.s.	n.s.
DPU		***	n.s.	***	n.s.	n.s.	n.s.
SFKU			**	***	**	**	**
SN				***	n.s.	n.s.	n.s.
MG					***	***	***
Tan						—	n.s.
Hu							n.s.
c.1040T>C (Phe347Ser)							
UM	n.s.	—	—	***	—	—	—
DPU		n.s.	n.s.	***	n.s.	n.s.	n.s.
SFKU			—	*	—	—	—
SN				***	—	—	—
MG					*	n.s.	n.s.
Tan						—	—
Hu							—
g.46544883A>G							

UM	***	n.s.	n.s.	n.s.	n.s.	n.s.	n.s.
DPU		*	**	***	n.s.	n.s.	n.s.
SFKU			n.s.	n.s.	n.s.	n.s.	n.s.
SN				n.s.	n.s.	n.s.	n.s.
MG					n.s.	n.s.	n.s.
Tan						n.s.	n.s.
Hu							n.s.

*UM: Ujimqin; DPU: Dorper × Ujimqin; SFKU: Suffolk × Ujimqin; SN: Sonid; MG: Mongolia; STH: Small—tailed Han; n.s.: Non—significant; *: $p < 0.05$; **: $p < 0.01$.
