# Supplementary material for: Effects of New Mutations in BMPRIB, GDF9, BMP15, LEPR, and B4GALNT2 Genes on Litter Size in Sheep
Source: Vet Sci. 2023 Mar 28;10(4):258. doi: 10.3390/vetsci10040258 (PMC10141719; doi:10.3390/vetsci10040258)
Supplement: Supplementary file 1 [file vetsci-10-00258-s001.zip › vetsci-2219749-supplementary/Supplementary Materials Table S1-S10/Table S8. BMP15 variant among eight sheep populations..pdf]

Table S8. Statistical significance for differences in the allele frequency of *BMP15* variant among eight sheep populations.

Variant/Breed	DPU	SFKU	SN	MG	Tan	Hu	STH
g.509807863G>A							
UM	***	*	***	***	n.s.	n.s.	n.s.
DPU		n.s.	n.s.	n.s.	n.s.	n.s.	n.s.
SFKU			n.s.	n.s.	n.s.	n.s.	n.s.
SN				n.s.	n.s.	n.s.	n.s.
MG					n.s.	n.s.	n.s.
Tan						—	—
Hu							—
c.31-33CTTinsdel (11Leu deletion)							
UM	***	n.s.	n.s.	n.s.	n.s.	n.s.	n.s.
DPU		***	***	***	**	***	*
SFKU			*	n.s.	n.s.	n.s.	*
SN				**	n.s.	n.s.	n.s.
MG					n.s.	n.s.	*
Tan						n.s.	n.s.
Hu							n.s.
c.755T>C (Lue252Pro)							
UM	n.s.	n.s.	*	n.s.	n.s.	n.s.	*
DPU		n.s.	n.s.	*	*	n.s.	*
SFKU			n.s.	n.s.	n.s.	*	*
SN				n.s.	*	n.s.	n.s.
MG					n.s.	n.s.	**
Tan						*	**
Hu							n.s.

*UM: Ujimqin; DPU: Dorper × Ujimqin; SFKU: Suffolk × Ujimqin; SN: Sonid; MG: Mongolia; STH: Small-tailed Han; n.s.: Non-significant; *: $p < 0.05$; **: $p < 0.01$.
