# Supplementary material for: Effects of New Mutations in BMPRIB, GDF9, BMP15, LEPR, and B4GALNT2 Genes on Litter Size in Sheep
Source: Vet Sci. 2023 Mar 28;10(4):258. doi: 10.3390/vetsci10040258 (PMC10141719; doi:10.3390/vetsci10040258)
Supplement: Supplementary file 1 [file vetsci-10-00258-s001.zip › vetsci-2219749-supplementary/Supplementary Materials Table S1-S10/Table S9. LEPR variant among eight sheep populations..pdf]

Table S9. Statistical significance for differences in the allele frequency of LEPR variant among eight sheep populations.

Variant/Breed	DPU	SFKU	SN	MG	Tan	Hu	STH
c.185G>A (Arg62His)							
UM	n.s.	***	*	***	n.s.	n.s.	n.s.
DPU		***	***	***	n.s.	*	n.s.
SFKU			*	***	**	n.s.	*
SN				***	n.s.	n.s.	n.s.
MG					*	***	***
Tan						n.s.	n.s.
Hu							n.s.
c.240C>T (80Asn)							
UM	***	n.s.	*	n.s.	n.s.	n.s.	n.s.
DPU		*	***	***	***	***	***
SFKU			n.s.	n.s.	**	n.s.	*
SN				n.s.	**	n.s.	*
MG					**	n.s.	*
Tan						n.s.	n.s.
Hu							n.s.
c.279C>T (93Ser)							
UM	***	n.s.	*	n.s.	n.s.	n.s.	n.s.
DPU		*	***	***	***	***	***
SFKU			n.s.	n.s.	**	n.s.	*
SN				n.s.	**	n.s.	*
MG					**	n.s.	*
Tan						n.s.	n.s.
Hu							n.s.

*UM: Ujimqin; DPU: Dorper × Ujimqin; SFKU: Suffolk × Ujimqin; SN: Sonid; MG: Mongolia; STH: Small-tailed Han; n.s.: Non-significant; *: $p < 0.05$; **: $p < 0.01$.
